# Editorial for Special Issue “Islet Transplantation”

**DOI:** 10.3390/cells14221810

**Published:** 2025-11-18

**Authors:** Shinichi Matsumoto

**Affiliations:** 1 Graduate School of Medicine, Kobe University, Kobe 650-0017, Japan; shinichi41@mac.com; 2 Medical Porcine Development Organization, Kobe 650-0017, Japan

Islet cell transplantation is revolutionary. Given recent developments, diabetes no longer seems incurable. Human islet cells have been approved by the FDA in the form of a drug named Lantidra^TM^, representing a breakthrough treatment for type 1 diabetes with severe hypoglycemia [1]. Clinical trials of embryonic stem cell (ESC)-derived islet transplantation have also been successful [2]. However, deriving ESCs from human fertilized eggs brings ethical issues [3]. To avoid this ethical issue, clinical trials of inducible pluripotent stem cell (iPS)-derived islet transplantation have been initiated in Asia, including in Japan [4].

The next major issue is the necessity of immunosuppressive drugs.

A clinical trial of encapsulated porcine islet xenotransplantation without immunosuppressive drugs has been initiated in the USA [5]. In Argentina, patients’ opinions 10 years after receiving encapsulated porcine islet xenotransplantation were very favorable [6], and similar outcomes are anticipated in the US clinical trial.

The ultimate goal of islet transplantation should be to enable type 1 diabetic patients to become insulin-independent without the need for life-long immunosuppressive drugs.

This goal can be achieved by inducing immunological tolerance and/or improving the efficacy of encapsulated islet xenotransplantation. Continuous research into islet cell transplantation will be important for achieving this goal.
